# Supervised vs. Self-Managed Exercise Therapy for Improving Shoulder Function After Traumatic Dislocation and Sprain: A Systematic Review and Meta-Analysis

**DOI:** 10.3390/sports13010023

**Published:** 2025-01-14

**Authors:** Daniel Koska, Robert Zetzsche, Tobias A. Mayer, Christian Maiwald

**Affiliations:** Institute of Human Movement Science and Health, Chemnitz University of Technology, 09126 Chemnitz, Germany; robert.zetzsche@hsw.tu-chemnitz.de (R.Z.); tobias.mayer@hsw.tu-chemnitz.de (T.A.M.); christian.maiwald@hsw.tu-chemnitz.de (C.M.)

**Keywords:** shoulder dislocation, functional status, exercise therapy, systematic review

## Abstract

Trauma-induced shoulder dislocations and sprains rank among the most common upper extremity injuries, with contact sports accounting for the majority of cases. These injuries often lead to substantial impairments in joint function and long recovery times, requiring targeted therapeutic interventions to restore mobility and prevent recurrent injuries. Given the pivotal role of exercise therapy in restoring shoulder function, this study systematically reviews the literature on the comparative effectiveness of supervised versus self-managed exercise therapy following acute shoulder trauma. PubMed, Cochrane CENTRAL, Embase, Web of Science, and Science Direct were searched up to 13 December 2024. Conservative and post-surgical treatment modes were analyzed separately. Five studies with a total 689 participants were included (conservative: *n* = 538 across two studies; post-surgical: *n* = 151 across three studies). Both treatment modes showed similar pooled effects (standardized mean difference, SMD_conservative_: −0.35, 95% CI [−1.39, 0.69]; SMD_post-surgical_: −0.23, 95% CI [−1.21, 0.75]), with a marginal improvement in shoulder function favoring supervised therapy. Four studies had some risk of bias, and one had serious risk; GRADE certainty was low. Supervised exercise therapy may offer slightly greater functional improvements over self-managed training, but evidence is limited by heterogeneity and low certainty. Further high-quality trials with standardized protocols and improved adherence tracking are needed to establish more definitive conclusions and guide clinical decision-making.

## 1. Introduction

Shoulder dislocations and sprains, classified under ICD-10 S43 [1], are common injuries resulting from trauma or excessive force that pushes the shoulder joint beyond its normal range of motion. High-impact sports activities—such as forceful overhead throwing motions in baseball, handball, or javelin, arm-pulling tackles in rugby or judo, and high-speed falls during skiing or cycling—are particularly prone to these injuries. Robinson et al. [2], in a study with 252 patients (15–35 years), found that contact sports was the most common cause of traumatic shoulder dislocation (86%). Correspondingly, Shah et al. [3] observed the highest incidence of 80.5 per 100,000 person-years observed in young men aged 16–20 years.

The management of traumatic shoulder injuries, including dislocations, depends on several factors such as a patient’s age, activity level, associated injuries, risk of recurrence, and the specific characteristics of the dislocation. In athletic populations, timely and effective management is critical not only for restoring function but also for enabling a quick and safe return to sport. Treatment may involve conservative measures, surgical intervention, or a combination of both. Regardless of the chosen approach, rehabilitation through structured physical exercise therapy appears to be essential for restoring shoulder function, including mobility, strength, and joint stability.

Exercise therapy can be delivered as either supervised or self-managed sessions, each offering distinct advantages and challenges that may affect recovery outcomes and healthcare costs. Supervised therapy refers to structured exercise programs conducted under the direct supervision of healthcare professionals, such as physiotherapists, in clinical settings. These professionals provide immediate, individualized feedback and hands-on guidance to patients, helping optimize recovery by addressing factors such as movement quality, muscle activation patterns, and joint stability. Controlled shoulder loading, in particular, has been shown to improve healing [4]. However, supervised therapy can be limited by logistical constraints, such as scheduling delays and a fixed number of prescribed sessions.

In contrast, self-managed therapy involves exercise programs performed independently by patients in non-clinical environments, such as at home. These programs offer greater flexibility, allowing patients to start training immediately after prescription and continue without restrictions on the number of sessions. This may contribute to improved outcomes, such as reduced shoulder pain and a faster return to activity [5]. Traditionally supported by written instructions, self-managed programs now increasingly incorporate video guidance and telehealth platforms. The growing integration of digital tools has further expanded the feasibility and appeal of self-managed approaches, making them increasingly relevant in modern rehabilitation practices [6,7].

For related shoulder conditions such as rotator cuff tendinopathy, adhesive capsulitis, and subacromial impingement syndrome, studies have shown comparable effectiveness between supervised and self-managed therapy [8,9,10]. However, for traumatic injuries classified under ICD-10 S43, no systematic review has yet synthesized evidence on the comparative effectiveness of these approaches for improving shoulder function. This review and meta-analysis aims to fill this gap, providing evidence-based guidance to clinicians and patients and advancing rehabilitation strategies for individuals recovering from acute shoulder injuries.

## 2. Materials and Methods

This review adhered to the methodological recommendations of the Cochrane Collaboration Handbook [11] and was conducted according to the Preferred Reporting Items for Systematic Reviews and Meta-Analyses (PRISMA) statement [12]. A review protocol for this study was registered on the PROSPERO database (ID 594587).

### 2.1. Search Strategy

The following databases were searched to identify relevant studies: MEDLINE via PubMed, the Cochrane Central Register of Controlled Trials (CENTRAL) via the Cochrane Library, Embase via Elsevier, Web of Science, and Science Direct. Papers published between 1990 and 2024 were included, with the most recent search conducted on 13 December 2024. The search strategy was initially developed for MEDLINE (see Appendix A) and subsequently adapted for the remaining databases. Additionally, after completing the full-text screening of articles identified through the database search, snowballing techniques, specifically reference list screening and citation tracking in Google Scholar, were applied to ensure comprehensive inclusion of relevant studies.

### 2.2. Study Selection

Titles and abstracts of the retrieved articles were independently evaluated by two reviewers (DK, RZ). Articles deemed relevant were retrieved in full-text for further assessment. Disagreements were resolved by consensus, and if unresolved, a third reviewer (TAM or CM) was consulted to assist with the final decision on article inclusion. A custom R [13] function was written to filter duplicates [14]. Careful examination confirmed that none of the included papers were multiple reports of the same study.

The applied eligibility criteria are presented in Box 1. Regarding the distinction between modes of therapy delivery, the following definitions were applied: Supervised therapy refers to exercise programs delivered with direct, in-person supervision by healthcare professionals, such as physiotherapists, typically conducted in clinical or other controlled environments. In contrast, self-managed therapy involves programs prescribed by healthcare professionals but performed independently by patients in non-clinical settings (e.g., at home). These programs are supported through written, oral, or video instructions provided via materials such as brochures or telehealth platforms. For more details on protocols and criteria distinguishing supervised and self-managed interventions, see Box 1.

Box 1 **Eligibility criteria.**

1. Participants: Studies involving patients with orthopedic conditions classified under ICD-10 S43. Conditions classified under other ICD-10 codes, such as rotator cuff injuries and tendinopathies (M75), shoulder impingement syndrome (M75.4), frozen shoulder (M75.0), fractures (S42), and thoracic outlet syndrome (G54.3), were excluded due to their distinct underlying mechanisms, which could lead to differential responses to specific interventions and introduce other confounding effects. Studies involving patients with recurrent anterior shoulder instability were eligible if they addressed both primary traumatic and recurrent anterior shoulder dislocations. Participants were required to be free from comorbidities that could potentially distort the study results, such as severe cardiovascular diseases, cancer, diabetes, Parkinson’s disease, or severe cognitive impairments (e.g., dementia). The mechanism of injury (e.g., sports injury vs. other causes) was not a criterion for inclusion.
  Only participants over 12 years of age were included to ensure generalizability to adolescent and adult populations and the validity of the outcome measures used. No upper limit was set for the maximum age of participants since no clear threshold could be identified at which age-related changes in shoulder function or recovery trajectories would render the interventions irrelevant or ineffective.
2. Interventions: Studies were included if at least one group performed self-managed exercises without professional supervision. Exercises could be administered using various methods, including video consultations, mobile applications, exergames, or conventional materials like brochures. Groups receiving only advice were also eligible for inclusion. In both the intervention and control groups, studies were excluded if they involved non-standard exercise therapies, such as blood flow restriction training or the use of movement monitoring devices (e.g., accelerometers). Studies were excluded if patients received additional treatments that could confound the effects of exercise, such as injections or local anesthesia. Studies involving surgical procedures were included if all patients across treatment arms underwent the same surgery, provided this did not involve shoulder joint replacement.
3. Comparators: Relevant comparisons included groups receiving standard supervised exercise therapy or assigned to a wait-list control, with all other conditions kept consistent with those outlined for the intervention group. Non-exercise therapies not typically performed at home, such as extracorporeal shockwave therapy or electrical stimulation, were excluded. Studies that blended supervised and self-managed components were excluded unless one mode clearly predominated. Predominance was determined by the vast majority of the intervention’s sessions, characteristics, or primary focus as described in the study design.
4. Outcomes: Included studies reported quantitative information on functional status using either validated Patient-Reported Outcome Measures (PROMs) questionnaires or validated performance-based tools. Studies were excluded if they only reported outcomes not directly reflecting shoulder function, such as structural parameters (e.g., cartilage thickness, bone mineral density), physiological variables (e.g., muscle atrophy), psychosocial variables (e.g., patient satisfaction), or other metrics like adherence, costs, and return-to-play times. Adherence monitoring was not a criterion for inclusion.
5. Study Type: Included studies were randomized controlled trials (RCTs) and controlled non-randomized studies (NRSs). Studies with low levels of evidence, such as case reports, were excluded. Additionally, non-clinical studies—including editorials, expert opinions, qualitative research, preclinical studies, and study protocols—were excluded, as well as reviews and meta-analyses. Studies including non-human models were excluded.
6. Study Duration: Included studies reported outcomes for interventions with a duration of at least four weeks.
7. Language: Publications in English or German were considered.
8. Year of publication: Studies published from 1990 to the present were included.

To determine eligibility for synthesis, the extracted characteristics of each study (e.g., intervention type, dosage, duration, and comparators) were tabulated and compared against the predefined groupings outlined above. Additional verification included checking alignment with the review’s objectives and ensuring consistency in outcome measures. Discrepancies in study categorization were resolved through consensus between two reviewers (DK, RZ).

### 2.3. Data Extraction

One reviewer (DK) manually extracted data from the included studies using a pre-defined data extraction sheet (Table 1). Data extraction further included information relevant for the risk of bias assessment, such as randomization methods, blinding, adherence (including compliance rates and deviations from the intended intervention), missing data, and the availability of a review protocol (see Section 2.4). In addition, details on adverse events or complications were recorded. A second reviewer (RZ) double-checked the extracted data. Any disagreements were resolved through discussion.

### 2.4. Risk of Bias Assessment

To assess the risk of bias in the included studies, a two-pronged approach was employed, reflecting the differing methodologies of the studies. For RCTs, the revised version of the Cochrane Risk of Bias (RoB 2) scheme was utilized [20]. This tool evaluates key domains including the quality of the randomization process, adherence to the assigned therapy, handling of missing outcome data, selective reporting of outcomes, and the analysis approach (i.e., intention-to-treat versus per-protocol).

For NRS, the Risk of Bias in Non-Randomized Studies—of Interventions (ROBINS-I) tool was used [21]. The ROBINS-I tool is a comprehensive framework developed by the Cochrane Collaboration to specifically assess the risk of bias in studies where participants are not randomly allocated to interventions. This tool evaluates bias across seven key domains: confounding, selection of participants, classification of interventions, deviations from intended interventions, missing data, measurement of outcomes, and selection of the reported result. Based on the assessment of these domains, an overall judgment of bias is made, categorizing the study as having a low, moderate, serious, or critical risk of bias.

For both RCTs and NRSs, discrepancies in the evaluation were resolved through discussion between the two reviewers (DK, RZ).

### 2.5. Data Synthesis and Analysis

A meta-analysis was conducted to pool the effects across different studies. Conservative-only treatments and post-surgical interventions were analyzed separately. All statistical analyses were conducted in R [13] within RStudio [22]. The R package *esc* [23] was used to calculate weights and effect sizes for each study. These were then pooled using a random-effects model from the R package *meta* [24], which accounted for both within- and between-study variability.

Effect sizes were measured using standardized mean differences (SMDs) between assessment outcomes at the follow-up time:

$$SMD=self-managed_{follow-up}-supervised_{follow-up},$$

For calculating SMDs, mean values and their corresponding standard deviations (SDs) were used. In one of the included studies [15], no SDs were reported at follow-up. The author was contacted via email for additional information, but no response was received. Consequently, the missing values were imputed using the baseline SDs. This decision is supported by evaluations of SDs from various studies on physiotherapeutic interventions for shoulder conditions, including those in our meta-analysis, which showed no systematic changes in variability between baseline and follow-up outcomes. In another study [19], only range values were reported alongside mean scores. In this case, SDs were estimated using the procedure proposed by Walter and Yao [25].

Follow-up values at 12 weeks were prioritized, as 12 weeks represents a typical timeframe for assessing the impact of therapy; otherwise, the closest available time point was selected. PROMs were sign-corrected to ensure higher scores consistently indicated better outcomes in the self-managed group. For studies with multiple functional outcomes, the score most relevant to shoulder function was selected, prioritizing frequently reported measures to reduce heterogeneity. For an overview of all available functional outcomes at various time points in the included studies, see Table A1 in Appendix B.

The effects of the meta-analysis are graphically depicted in forest plots, showing SMDs, 95% confidence intervals, weights, and a pooled mean effect estimator.

#### 2.5.1. Heterogeneity

Heterogeneity was addressed through the random-effects model, with the between-study variance ($\tau^{2}$) representing heterogeneity in absolute terms and the proportion of variability attributable to heterogeneity ($I^{2}$) in relative terms. Subgroup analyses based on age groups were intended but could not be conducted due to insufficient data.

A sensitivity analysis was conducted for one of the two treatment modes (post-surgical exercise therapy; see Appendix C). For the other mode (conservative only treatment), only *n* = 2 studies were available, which precluded a meaningful sensitivity analysis.

#### 2.5.2. Threshold for Clinically Important Effects

To assess the relevance of the pooled mean effects, the minimal important differences (MIDs) for the outcome scores included in the meta-analysis were standardized and averaged. Specifically, each MID (Constant–Murley Score (CMS) = 8.3 [26], Oxford Shoulder Instability Score (OSIS) = −6.0 [27]) was divided by the SDs of the respective scores in the study population (CMS = 18 [15], OSIS = 10.9 [16]).

The resulting standardized MIDs for the CMS and OSIS were 0.46 and −0.55, respectively. Since no MID could be determined for the Functional Impairment Test-Hand and Neck/Shoulder/Arm [28] (FIT-HaNSA), we assumed a standardized MID equal to the average of the values for OSIS and CMS (±0.5). This assumption is supported in Norman et al. [29], who showed that the MIDs of health-related PROMs are typically close to half a SD.

### 2.6. Summary of Evidence

The quality of evidence of the included studies was assessed using the Grading of Recommendations Assessment, Development, and Evaluation (GRADE) scheme [30]. GRADE evaluates five aspects: study limitations (risk of bias), inconsistency of results (heterogeneity), indirectness of evidence, imprecision of the effect estimates, and reporting bias. Based on these criteria, the quality of the evidence was classified as high, moderate, low, or very low.

## 3. Results

### 3.1. Study Selection

**Figure 1:**
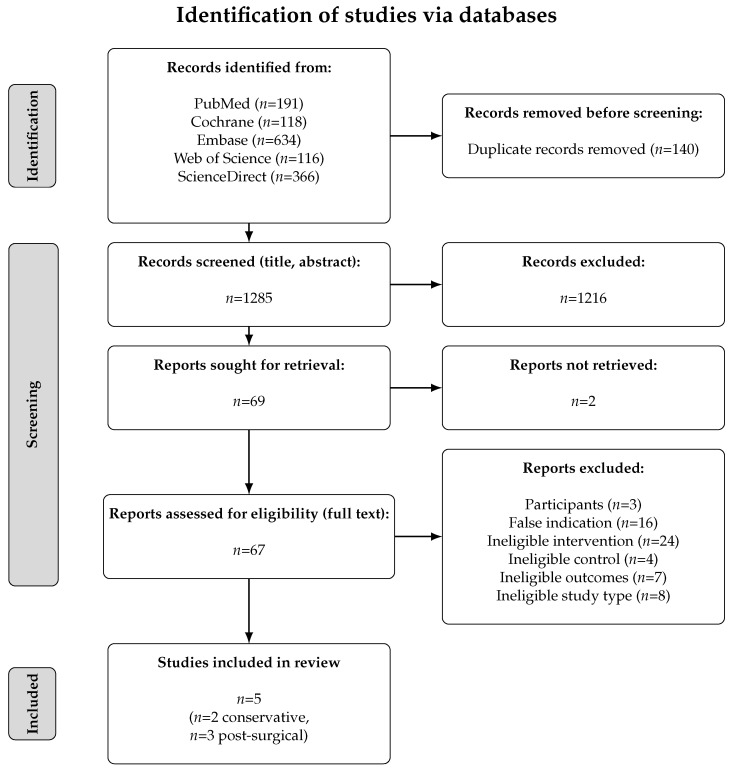
Identification of studies via databases (PRISMA flow diagram).

Our search resulted in 1425 hits across all databases. A total of 140 hits were identified as duplicates, resulting in 1285 titles and abstracts to be screened according to the eligibility criteria defined in Section 2.2. From these, 67 papers remained for a full-text review. Of these, five studies were ultimately included. An overview of the entire screening process including a breakdown by exclusion criteria is presented in Figure 1.

### 3.2. Study Characteristics

Table 1 provides a summary of the characteristics of the studies included in this review. Five trials were analyzed, consisting of four RCTs and one NRS, published between 2014 and 2024. These studies collectively involved 689 participants and assessed a range of functional outcomes. An overview of all available functional outcome scores across all follow-up times is presented in the Appendix B (Table A1).

#### 3.2.1. Conservative Treatment

Eshoj et al. [15] investigated participants with radiographically verified, trauma-initiated primary or recurrent anterior shoulder dislocation and self-reported decreased ability to perform shoulder movements during daily activities in the previous 7 days, with no history of previous surgery. Participants were randomized to either a home-based (HOMEX) or a supervised exercise group (SINEX). The HOMEX group received an introductory session and performed exercises independently at home, while the SINEX group attended tailored, supervised sessions. For SINEX, an attendance of at least 50% of the supervised sessions (7 of 14 possible) was required in addition to completion of at least two-thirds of the planned HOMEX exercises (self-reported training diary).

Kearney et al. [16] investigated participants who had a first-time traumatic anterior shoulder dislocation confirmed radiologically and managed non-operatively. Participants were randomized into two groups: An advice group receiving a single session of advice on shoulder management, including educational materials and the option to self-refer to physiotherapy, and an advice + physiotherapy group receiving the same advice plus a structured physiotherapy program. The physiotherapy program involved progressive exercises designed to restore shoulder function, improve range of motion, and strengthen shoulder stabilizers. Intervention fidelity was monitored using direct observations, audio recordings, and physiotherapy checklist self-reports.

#### 3.2.2. Post-Surgical Treatment

Eren et al. [17] investigated participants diagnosed with recurrent anterior shoulder instability who underwent an arthroscopic capsulolabral (Bankart) repair following four weeks of immobilization. Participants were divided into either a home-based rehabilitation group or a hospital-based rehabilitation group. Both groups followed an identical rehabilitation protocol focused on restoring shoulder range of motion and strength, with the home-based group performing exercises independently and the hospital-based group receiving supervised physiotherapy sessions. Patients in the home-based rehab group were instructed to fill out an exercise checklist daily and were called for follow-up every three weeks, while patients in the hospital-based rehab group attended therapy sessions three times weekly.

Ismail et al. [18] investigated participants who had recurrent anterior shoulder instability treated with arthroscopic anterior shoulder stabilization using suture anchors and capsular shift. Participants were randomly assigned to either a supervised or a home-based rehabilitation group. The supervised group attended three sessions per week for 24 weeks at an outpatient clinic, where adherence was monitored through attendance records. In contrast, the home-based group followed a similar program independently, with guidance provided only at the beginning of each phase, and had the option to consult the therapist as needed.

Martinez-Rico et al. [19] investigated participants who underwent arthroscopic Bankart repair for recurrent anterior shoulder instability. Patients were randomly assigned to one of two groups: a control group performing unsupervised at-home exercises and a study group performing the same exercises but receiving additional support through thrice-weekly coaching phone calls from a nurse who had access to a physiotherapist. Before beginning home training, all participants received about three weeks of supervised physiotherapy at an outpatient clinic.

### 3.3. Risk of Bias

The assessment of the risk of bias was conducted separately for RCTs and the NRS. This section starts with the results for the RCTs, then follows up with the included NRS.

#### 3.3.1. RCT

The risk of bias was consistent across all included RCTs (Table 2). All studies exhibited some concerns in two domains:

1. Bias due to deviations from intended interventions: In all included RCTs, both participants and caregivers were aware of their assigned group. This issue is inherently linked to the study design, where complete blinding of participants is rarely achievable.
2. Bias in measurement of the outcome: The use of subjective measures to quantify functional outcomes in all but one study may potentially affect the reliability of the results.

Publication bias was not formally assessed due to the limited number of included studies. However, additional aspects regarding the risk of bias were identified in each of the studies:

In Eshoj et al. [15], the risk of bias mainly arises from the use of different exercise concepts in the two treatment arms. The SINEX group received neuromuscular shoulder exercises, while the HOMEX group primarily engaged in strength training. Moreover, variability in compliance was observed, with only 43% of the SINEX group and 54% of the HOMEX group meeting the predefined thresholds for supervised sessions and home-based exercises. This variability raises concerns that the full intended effect of the interventions may not have been consistently delivered across participants. No substantial deviations were identified between the pre-registered protocol (NCT02371928) and the published study.

Concerns in the study by Kearney et al. [16] resulted primarily from the lack of a pure comparison between supervised (physiotherapy) and self-managed care (advice only). The supervised group received the same treatment as the advice only group, plus additional physiotherapy, leading to a blending of treatment modalities. Moreover, participants in the advice-only group were allowed to self-refer to physiotherapy, which may have further blurred the distinction between the groups. In addition, 27% of participants were lost to follow-up. The authors, however, conducted a sensitivity analyses to analyze the effect the missing data, which suggested that this did not introduce significant bias into the study’s findings. No important deviations between the pre-registered protocol (ISRCTN63184243) and the published study were found.

The study by Ismail et al. [18] raised additional concerns of bias resulting from the measurement of the outcome. The study used the FIT-HaNSA score as the primary outcome measure. Unlike the PROMs used in the other studies, the objective FIT-HaNSA test focuses on specific tasks that may not fully capture all dimensions of shoulder or upper limb function, potentially limiting the comprehensiveness of the assessment. No publicly available pre-registered protocol was identified for this study.

Further concerns of bias in the study by Martinez-Rico et al. [19] stem from differences in adherence tracking between the groups: adherence was actively monitored through regular phone calls in the supervised group, while no adherence monitoring was reported for the self-managed group, potentially leading to unequal levels of engagement with the interventions or performance bias. The study also incorporated a combination of supervised and unsupervised training modalities, with all participants engaging in both conventional, supervised rehabilitation and independent, unsupervised home exercises, which could potentially introduce variability in the treatment’s application and effectiveness. No publicly available pre-registered protocol was identified for this study.

#### 3.3.2. NRS

The included NRS [17] indicated a serious risk of bias in two domains: The first major risk factor is the non-randomized allocation of participants to either the self-managed (home-based) or supervised (hospital-based) rehabilitation groups (Table 3). This allocation, based on patient preference, could have introduced baseline differences between the groups—such as variations in motivation, health status, or other unmeasured factors—that were not controlled in the analysis (bias due to confounding). Secondly, the risk of bias was considered serious due to substantial variability in adherence between groups; the home-based group showed lower adherence to the prescribed rehabilitation protocol, raising concerns that the full intended effect of the interventions may not have been consistently achieved (bias due to deviations from intended interventions).

Additionally, the study was judged to have a moderate risk of bias in the domain bias in selection of participants for the study, as self-selection into the treatment groups could result in differences in characteristics like motivation, time availability, or comorbidities, potentially affecting outcomes. Similar to the included RCTs, the study was assigned a moderate risk of bias in the domain bias in measurement of outcomes due to the use of (subjective) PROMs and the lack of blinding, which could have influenced self-reported outcomes. No publicly available pre-registered protocol was identified for this study.

### 3.4. Intervention Effects

#### 3.4.1. Conservative Treatment

**Figure 2:**
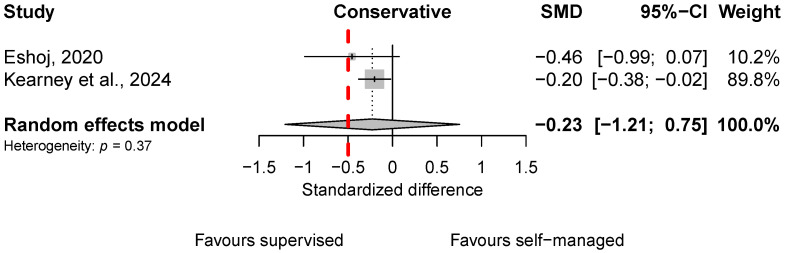
Forest plots of standardized mean differences (SMDs) in shoulder function between supervised and self-managed exercise therapy for post-surgical treatment. The red line indicates the threshold for clinically important differences. Included scores (see Table 1): CMS [15] and OSIS [16].

Two trials analyzed participants treated entirely conservatively [15,16] (Figure 2). The pooled mean effects showed a slightly larger functional improvement in the supervised group in both the conservative and post-surgical treatment condition (Figure 2). This effect was neither statistically significant nor clinically relevant (see the red line in Figure 2) and was accompanied by substantial uncertainty.

#### 3.4.2. Post-Surgical Treatment

**Figure 3:**
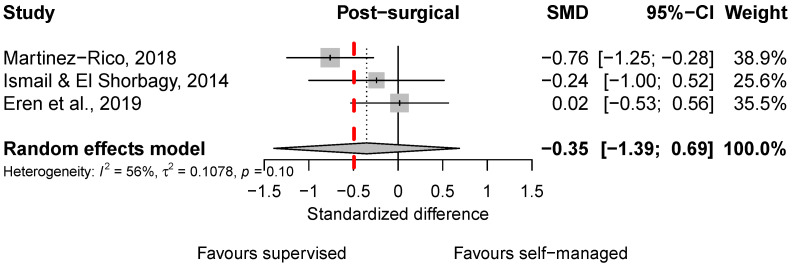
Forest plots of standardized mean differences (SMDs) in shoulder function between supervised and self-managed exercise therapy for post-surgical treatment. The red line indicates the threshold for for clinically important differences. Included scores (see Table 1): OSIS [19], CMS [17], and Fit-HaNSA [18].

Three studies analyzed participants treated post-surgically [17,18,19] (Figure 3). The pooled mean effect was similar in size and direction to that observed for conservative treatment (Figure 3) and failed to reach statistical significance and clinical importance. Heterogeneity was substantial (*I*^2^ = 56%) with an estimated between-study variance of (tau^2^ = 0.11), indicating notable variability in effect sizes across the included studies.

The quality of the evidence in both scenarios (conservative, post-surgery) was rated as low (Table 4).

### 3.5. Adverse Events

#### 3.5.1. Conservative Treatment

Eshoj et al. [15] stated that only a few participants reported recurrent anterior shoulder instability or subluxations at the 12-week follow-up, with no significant difference between groups. Short-term adverse events were infrequent and did not differ significantly between groups. The most common adverse events reported were exercise-induced shoulder pain, soreness, and muscle fatigue, occurring equally in both the supervised and self-managed groups (*n* = 10 each). However, three participants in the supervised group (neuromuscular shoulder exercises) reported an increase in shoulder pain, necessitating a temporary modification of their exercise program.

Kearney et al. [16] reported a total of 41 adverse events in the advice-only group and 35 in the advice and physiotherapy group. These events included rotator cuff tears, shoulder re-dislocations, frozen shoulder, compression fractures, and ongoing nerve damage. The overall incidence of adverse events was comparable between the two groups, with 15.8% of participants experiencing at least one complication. No statistically significant differences were observed in the complication rates between the treatment arms.

#### 3.5.2. Post-Surgical Treatment

Eren et al. [17] reported two adverse events (recurrent dislocations), both unrelated to the exercise therapy intervention and occurring well after the analyzed intervention period of three months (at 24 and 17 months after surgery).

Ismail et al. [18] reported that no serious adverse events occurred during the study period in either the supervised or the home-based rehabilitation groups. The study did not document any recurrent dislocations or significant differences in adverse events between the groups throughout the study period.

The study by Martinez-Rico et al. [19] reported no surgical complications, no need for surgical revision, and no recurrent dislocations during the study period. All participants in the study group completed the phone contact program. One participant from the unsupervised home exercise group did not complete the follow-up due to psychiatric admission for schizophrenia and was excluded from the analysis. No other adverse events or complications were reported.

## 4. Discussion

This systematic review and meta-analysis synthesized data from five studies comprising a total of 689 participants with shoulder dislocations and sprains, examining the effects of supervised versus self-managed exercise therapy across two treatment modalities: conservative and post-surgical. The results indicate that supervised exercise therapy may yield slightly better shoulder function improvements compared to self-managed therapy. The overall quality of evidence was rated as low, with substantial uncertainty surrounding the findings. Moreover, neither of the pooled mean effects in either treatment condition met the assumed clinical relevance threshold of ±0.5 SDs (SMD_conservative_: −0.35, 95% CI −1.39 to 0.69; SMD_post-surgical_: −0.23, 95% CI −1.21 to 0.75). Therefore, the current evidence remains insufficient to establish the superiority of one treatment approach over the other.

Transferability to a sports context

Although this review did not exclusively target sports injuries, its broader scope ensured a comprehensive dataset and minimized the risk of missing relevant studies. Despite this, its findings remain highly transferrable to a sports context for several reasons:

First, the therapeutic principles underlying exercise-based interventions address the demands of the injury itself (e.g., restoring joint stability, function, and strength) rather than the specific circumstances of the injury. Moreover, no clinical evidence suggests fundamental differences in recovery trajectories between sports-related injuries and those from other contexts. Second, the age profiles of participants in the included studies suggest a strong overlap with athletic populations, particularly those engaged in high-risk activities such as contact sports. Apart from Kearney et al. [16], who included a broad age range of adults (mean age 44.9 [19.6] years), the included studies included mostly younger individuals, with mean ages ranging from 25.8 (5.8) to 30.5 (9.1) years. These demographics are consistent with populations frequently involved in sports at heightened risk of traumatic shoulder injuries.

Alignment with existing evidence

The outcome of this review is partly consistent with similar systematic reviews on other shoulder conditions. Gava et al. [9] reported very-low-certainty evidence suggesting no significant difference between home-based exercise programs delivered via telerehabilitation and in-person physical therapy for reducing pain and improving disability in individuals with shoulder pain. Similarly, Littlewood et al. [8] found that whether exercise is performed at home or in a clinic setting does not seem to affect outcomes for rotator cuff tendinopathy, although uncertainty remains regarding key prescription parameters, such as exercise type, number of sets, level of resistance, or the optimal number of repetitions. Gutierrez-Espinoza et al. [10] provided moderate-quality evidence indicating that supervised physiotherapy and home-based exercise are similarly effective for patients with subacromial impingement syndrome. Zhang et al. [31] reviewed studies on the effectiveness of telemedicine for patients with rotator cuff disorders and found that telemedicine significantly improved shoulder function, as measured by the CMS and QuickDASH, while also reducing pain and improving range of motion compared to conventional in-person rehabilitation programs. Likewise, Huang et al. [32] conducted a systematic review and meta-analysis comparing telerehabilitation and home-based exercises for various shoulder conditions, including subacromial impingement syndrome, shoulder stiffness, and rotator cuff tears. They found low-to-moderate-quality evidence that telerehabilitation significantly improved range of motion, functional outcomes, and quality of life compared to home-based exercises.

These studies suggest that self-managed training delivered via digital communication technologies may offer distinct advantages over traditional approaches for delivering self-managed exercise therapy, such as informational booklets or initial advice sessions. The present review, however, did not identify any studies comparing telerehabilitation programs to other therapeutic modalities for acute shoulder dislocations and sprains. The transferability of this finding to the diseases investigated in this study is therefore limited and should be a focus of future research efforts.

Heterogeneity

Compared to the evidence available for other shoulder conditions, and despite the high prevalence of shoulder dislocations and sprains [3,33], only few high-quality studies have compared supervised and self-managed exercise therapy for patients classified as ICD-10 S43. This scarcity of high-quality studies limits the strength of the pooled effect estimates and amplifies the influence of methodological differences or variations in patient populations across studies.

For post-surgical treatment, the *I*^2^ value of 56% indicates that more than half of the variability in effect estimates is due to heterogeneity rather than chance, reflecting moderate to substantial inconsistency among study results. For instance, Martinez-Rico et al. [19] report a statistically significant and clinically relevant effect favoring supervised therapy, whereas Eren et al. [17] suggest nearly identical outcomes between supervised and self-managed approaches. In the conservative treatment condition, an *I*^2^ value could not be calculated due to the limited number of studies (*n* = 2). Nevertheless, both studies report results trending in the same direction, favoring supervised therapy over self-managed approaches. The small number of studies, however, highlights the need for more comprehensive research to confirm these findings and strengthen the evidence base.

Sources of heterogeneity

Heterogeneity is evident in the differences across exercise modalities, outcome measures, and participant characteristics, including sex. Participants in the supervised and self-managed groups, for instance, did not follow the same exercise protocols in all studies: Eshoj et al. [15] included a home-based group that followed a standard care program focused mainly on muscle strengthening exercises, while the supervised physiotherapy group participated in a regimen aimed at enhancing movement quality through neuromuscular exercises targeting balance, coordination, strength, and proprioception. In Ismail et al. [18], the home-based group began with three weeks of absolute immobilization in a sling, whereas the supervised group started with movement exercises (e.g., unweighted pendulum exercise) immediately after surgery. In Kearney et al. [16], the control group received only advice and materials, without a structured exercise program, while the intervention group followed an individualized physiotherapy program in addition to advice and supporting materials. This also highlights that some studies also included an overlap of different training modalities: In Eshoj et al. [15], the supervised group had additional access to online exercise instructions and videos through a web-based platform. In Martinez-Rico et al. [19], all participants underwent the same supervised physiotherapy program before transitioning to unsupervised or guided home-based exercises. Such overlap could blur the distinction between intervention groups, complicating efforts to isolate the effects of each program and potentially favoring supervised therapy.

Variability is also evident in how self-managed training was delivered across studies. In Kearney et al. [16], participants received advice-only sessions supplemented by web-based materials and a trial intervention manual. Eshoj et al. [15] incorporated an introductory supervised physiotherapy session, complemented by a leaflet with photographs and descriptions of exercises. Martinez-Rico et al. [19] employed a phone-assisted nursing program to support self-management. Ismail et al. [18] provided patients with phase-specific training sessions accompanied by written instructions, illustrations, and advice. Eren et al. [17] instructed participants to complete daily exercise checklists and conducted follow-up calls every three weeks. These diverse training delivery methods likely influenced outcomes in unique ways, although their distinct impacts remain difficult to quantify.

Furthermore, the outcome measures vary across studies, contributing to the variability in the results. While scores like the OSIS specifically assess shoulder instability and function, other scores, such as the CMS and FIT-HaNSA, provide broader evaluations of upper extremity function or specific daily activities. Notably, the CMS and FIT-HaNSA are not pure PROMs: the CMS includes both subjective components and objective measurements, such as range of motion and strength [34]. The FIT-HaNSA, in particular, is a functional performance test that simulates daily activities. It was included in the meta-analysis since its tasks closely mirror real-life shoulder function demands, and its test scores were shown to correlate considerably with PROMs such as the DASH (r=−0.76) and SPADI (r=−0.71) in patients with shoulder disorders [35].

In addition to the previously mentioned points, there was some heterogeneity in sex across the included studies, with a male predominance across all studies, ranging from 66% [16] to over 90% in other studies. On the one hand, the frequent inclusion of younger male participants simply reflects the epidemiology of shoulder dislocations. Shah et al. [3] reported that 72% of shoulder dislocations occurred in men, with the highest incidence among those aged 16–20 years (80.5 per 100,000 person-years). On the other hand, shoulder dislocations and sprains of the shoulder girdle’s joints and ligaments are not confined to younger males. Shah et al. [3] identified an (unexpected) increase in the incidence among women over 50 years, a pattern not observed in men. Also, men and women differ in relevant features such as shoulder anatomy, muscle strength, ligament laxity, and hormonal influences, all of which can affect injury patterns, healing rates, and responses to rehabilitation. Therefore, findings from studies predominantly involving younger male participants may be limited in their ability to generalize. This highlights the necessity for more balanced and representative research to better understand the relative effectiveness of different rehabilitation strategies across diverse patient populations.

All these factors, along with others not discussed in detail—such as cultural and socioeconomic factors, rehabilitation duration and environment, and psychosocial aspects—contribute to the observed heterogeneity. To accurately attribute differences to the mode of delivery (supervised vs. self-managed), the most critical step, in our opinion, is ensuring that participants in different treatment groups receive consistent types and amounts of exercises. We recommend that future studies implement standard exercise protocols, such as those outlined in consensus guidelines (e.g., the American Society of Shoulder and Elbow Therapists’ Consensus Rehabilitation Guideline [36] or similar evidence-based frameworks), to ensure consistency and comparability across groups. These protocols typically follow a staged approach: initial protection of the repair and limited ROM, gradual progression of ROM and strength, and, finally, a focus on dynamic stability and functional activities in later phases. Regardless of the exact design of the exercise therapy, ensuring comparable treatments across groups remains essential to attribute differences in outcomes to the mode of delivery.

Conservative vs. post-surgical treatment

A key factor influencing the effectiveness of exercise therapy across different populations is their status prior to starting therapy—whether they are treated post-surgically or entirely conservatively. Conservatively treated patients often have less severe injuries to begin with, less tissue disruption, and different pain or inflammation profiles, allowing for more flexibility in exercise progression. In contrast, post-surgical patients typically require adherence to specific healing timelines, restricted movements, and a gradual rehabilitation protocol, as surgery often involves tissue repair or reconstruction. These factors may lead to the expectation that supervised programs would be particularly beneficial for post-surgical patients, given their need for guidance in adhering to movement restrictions and ensuring proper technique. Conservatively treated patients, on the other hand, might seem better suited to self-managed programs due to their typically greater flexibility and lower risk. The results of this meta-analysis, however, challenge this expectation, showing a similarly directed effect across both treatment conditions. Further research is needed to determine whether this reflects an artifact of the low certainty of our findings or a genuine effect.

Limitations

This review includes a small number of studies, divided into two separate treatment conditions, limiting the statistical power and generalizability of the findings. Considerable heterogeneity was observed among the included trials. The risk of bias assessment indicated “some concerns” for the four RCTs, while a “serious risk” of bias was identified for the included NRS [17]. The elevated risk in the NRS is partly due to the non-randomized allocation of participants, since treatment arms were determined by participant preference rather than random assignment. This approach may have led to imbalances in baseline characteristics between groups, complicating the interpretation of comparative effectiveness. Notably, this was the only study in which our model did not suggest the superiority of supervised therapy.

Concerns have been raised regarding the revised Cochrane risk of bias tool [11] used in this study. Previous research has reported low inter-rater agreement for the overall risk of bias judgement (κ = −0.15, 0.16) and only moderate agreement for individual items, such as ’bias arising from the randomisation process’ (κ = 0.45) [37]. To address this and enhance reliability, we conducted an intensive training session and calibration exercise among all raters prior to the application of the tool.

Several other potential sources of bias were identified across the included studies, which may affect the interpretation of their findings. These include the lack of blinding of participants in all studies (a challenging aspect in these types of trials), modest sample sizes in all but one study, and unquantified publication bias. While publication bias was not formally assessed due to the limited number of included studies, a comparison of prespecified protocols with published results for two studies revealed no meaningful deviations. Consistency between methods and results was observed in the remaining studies, and additional searches in Google Scholar and clinical trial registries identified no unpublished completed trials, providing no indications of publication bias. Further issues were identified regarding the methods of monitoring or reporting adherence across studies:

Eshoj et al. [15] tracked adherence using self-reported training diaries. Among those returning diaries after completing the home exercise program (*n* = 18 per group), adherence was 71% in the supervised group (SINEX) and 79% in the self-managed group (HOMEX). In Kearney et al. [16], adherence in the advice-only group was 98%, with 81% of participants completing the program as advised and 18% self-referring for physiotherapy. In the supervised group, 69% completed all sessions, while 10% did not attend any additional appointments, and 12% stopped attending after one session. Martinez-Rico et al. [19] implemented structured adherence tracking in the supervised group through regular coaching phone calls but did not report specific adherence rates. Ismail et al. [18] tracked adherence in the supervised group through attendance at scheduled sessions, whereas no formal tracking was implemented for the self-managed group. Eren et al. [17] directly monitored adherence in the hospital-based group via session attendance, with an average of 13.8 ± 7.3 sessions completed over six to eight weeks. In the home-based group, adherence was self-reported using daily exercise checklists and follow-up calls every three weeks, but specific numbers of completed sessions were not provided.

Differences in adherence present a considerable challenge, as adherence is a well-documented confounder in rehabilitation studies [38]. DiMatteo et al. [39] reported the average outcome difference between high and low adherence as 26%. In particular, various studies indicate that home-based programs often have lower adherence rates than supervised therapy [40,41,42,43]. This may bias outcomes in favor of supervised therapy, as reduced adherence may result in poorer results [44,45]. In our results, the study by Martinez-Rico et al. [19] showed the largest effect in favour of supervised therapy. This may be partially explained by the phone-based follow-up calls in the supervised group, which could have introduced additional support and inadvertently biased results in favor of the intervention. Addressing adherence issues is therefore essential to accurately evaluate the relative effectiveness of supervised and self-managed rehabilitation strategies.

One way to address adherence in self-managed settings is through digital tools such as smartphone applications and telehealth platforms [46,47]. With features such as reminders, progress tracking, and remote monitoring by healthcare providers, these tools provide practical and effective strategies to enhance adherence and support better patient outcomes. Research into the impact of these tools is rapidly growing stimulated by circumstances such as the COVID-19 pandemic, or initiatives such as the Digital Healthcare Act in Germany [48]. Positive effects of digital tools have already been demonstrated for certain conditions (see the discussion under “Alignment with existing evidence”) and upcoming evidence is likely to increase the certainty of our findings and potentially influence their direction.

Lastly, this review intentionally focused on functional outcomes, as these are key indicators of success in exercise therapy, particularly in sports-related contexts. While this focus allowed for a detailed analysis of function, it inherently narrowed the range of outcomes considered. Factors such as health-related quality of life or intervention costs were not included to maintain the feasibility of this review, which already encompassed two distinct treatment modes (conservative and post-surgical). Future reviews should address these additional dimensions to provide a more comprehensive evaluation of therapeutic interventions.

## 5. Conclusions

This systematic review presents low-certainty evidence that patients with shoulder dislocations and sprains, whether treated conservatively or post-surgically, may experience slightly greater improvements in shoulder function with supervised therapy. Further rigorous research—including well-designed randomized controlled trials with standardized exercise across treatment groups, larger sample sizes, and adherence tracking—is needed to draw more definitive conclusions.

Emerging technologies, particularly digital health tools, are gaining research interest due to their potential to enhance adherence and accessibility in self-managed settings. As studies incorporating these tools increase, the evidence base is expected to expand, leading to greater certainty in findings. This, in turn, could better inform clinical decision-making and optimize rehabilitation strategies compared to the current evidence base.

## Figures and Tables

**Table 1:** Characteristics of included studies. Outcome scores used in the meta-analysis are marked with an asterisk.

Author (Year), Country, Design	Participants	Intervention		Outcome	
		Self-Managed	Supervised	Self-Managed	Supervised
Conservative Treatment					
Eshoj et al. (2020) [15], Denmark, Multicentre, superiority RCT	*n* = 56 Included age: 18–39 years Mean age = 25.8 (5.8) Ratio M/F: 49/7	HOMEX (Home-based exercise, *n* = 28): Single introductory supervised physical therapy session, including a leaflet with photographs and descriptions of exercises, active exercises for the rotator cuff and scapular muscles using elastic bands and one exercise for mobility/coactivation of the scapular and core stability muscles.	SINEX (Supervised exercise, *n* = 28): Individually tailored, supervised sessions of progressive shoulder exercise in addition to functional kinetic chain exercise, access to online exercise instructions and videos through the physical therapy website.	WOSI (total) Baseline: 1145.5 (376.2) 3 Months: 718.3 (NR) CMS (total) * Baseline: 67.6 (20.7) 3 Months: 80.6 (NR)	WOSI (total) Baseline: 970.2 (346.9) 3 Months: 314.9 (NR) CMS (total) * Baseline: 72.6 (15.3) 3 Months: 88.9 (NR)
Kearney et al. (2024) [16], UK, Pragmatic, superiority multicentre RCT	*n* = 482 Included age: ≥18 years Mean age = 44.9 (19.6) Ratio M/F: 317/165	Advice only (*n* = 240): Single session of advice, supporting materials, and option to self-refer to physiotherapy.	Advice + physiotherapy (*n* = 242): Additional programme of physiotherapy.	OSIS * Baseline: NR 3 Months: 30.0 (11.4) QuickDASH Baseline: NR 3 Months: 22.8 (21.7)	OSIS * Baseline: NR 3 Months: 32.2 (10.4) QuickDASH Baseline: NR 3 Months: 19.3 (19.9)
Post-surgical Treatment					
Eren et al. (2019) [17], Turkey, Non-randomized controlled trial	*n* = 54 Included age: ≥16 years Mean age = 30.5 (9.1) Ratio M/F: 49/5	Home-based (*n* = 33): Exercise program consisting of five phases (group allocation performed after the first phase): 1. maximum protection (e.g., isometric deltoid strengthening); 2. limited motion (e.g., passive ROM exercises with max 140° forward flexion); 3. medium protection (e.g., isotonic strengthening using dumbbells or Theraband); 4. minimum protection (e.g., closed kinetic chain exercises like push-ups); and 5. function phase (e.g., plyometric exercises).	Supervised hospital-based (*n* = 21):Note: Apart from being home or hospital-based, rehabilitation programs were constructed to be identical regarding the exercises. Postural exercises with sling and isometric exercises for deltoid strengthening were initiated on the day after surgery.	DASH Baseline: 27.46 (11.81) 3 Months: 7.93 (8.4) CMS * Baseline: 58.23 (14.23) 3 Months: 85.29 (14.02) Rowe Baseline: 51.72 (15.36) 3 Months: 86.79 (15.64)	DASH Baseline: 32.53 (16.42) 3 Months: 11.08 (11.41) CMS * Baseline: 54.17 (10.46) 3 Months: 85.03 (17.92) Rowe Baseline: 43.81 (19.16) 3 Months: 87.0 (12.04)
Ismail et al. (2014) [18], Egypt, Single-blinded RCT	*n* = 27 Included age: 18–35 years Mean age = 26.9 (7.3) Ratio M/F: 24/3	Controlled home-based (*n* = 13): A 24-week program divided into four phases, including mobility exercises, resistance training with elastic bands, and shoulder stabilization drills, with patients receiving initial instructions at the start of each phase.	Supervised (*n* = 14): A 24-week program with three supervised sessions per week at an outpatient clinic, including progressive resistance exercises, functional training, and manual therapy (program divided into four phases with progressive exercises).	FIT-HaNSA * Baseline: NR 3 Months: 240.3 (52.7)	FIT-HaNSA * Baseline: NR 3 Months: 251.5 (39.5)
Martinez-Rico et al. (2018) [19],Spain, Randomized controlled trial	*n* = 70 Included age: NR Median age = 26 (range = 18–46) Ratio M/F: 54/16	Control group (HB, *n* = 34): Unsupervised home exercises. Note: In addition to conventional supervised rehabilitation, all participants received verbal and written information about activities and exercises to be performed at home daily. Written information included a handout with instructions on home exercises and advice on upper body motion, such as limiting extreme movements or carrying weight in the hand.	Study group (SV, *n* = 36): Participants received the same treatment as those in the control group, with the addition of three weekly calls from a nurse, who had access to a physiotherapist, during the first month. The nurse asked about activities undertaken at home and provided additional coaching sessions about self-care, the importance of the exercises at home, instructions on performing the exercises, and responses to their questions.	DASH Baseline: 30.1 (31–58) 4 Months: 25.9 (9–86) OSIS * Baseline: 36.7 (24–53) 4 Months: 26.4 (12–51) Rowe Baseline: 40.0 (5–75) 12 Months: 89.1 (65–100)	DASH Baseline: 28.9 (2–54) 4 Months: 9.0 (0–36) OSIS * Baseline: 36.4 (25–53) 4 Months: 20.4 (12–36) Rowe Baseline: 42.6 (15–75) 12 Months: 93.4 (70–100)

Abbreviations: NR—Not Reported; M/F—Male/Female; Age—Mean (standard deviation) or median age (range) in years; OSIS—Oxford Shoulder Instability Score; (Quick) DASH—(Quick) Disabilities of the Arm, Shoulder, and Hand; WOSI—Western Ontario Shoulder Instability Index; CMS—Constant–Murley score.

**Table 2:** Risk of bias assessment as recommended by the Cochrane Collaboration Handbook [11]. Domains: D1: Bias arising from the randomization process; D2: bias due to deviations from intended interventions; D3: bias due to missing outcome data; D4: bias in measurement of the outcome; and D5: bias in selection of the reported result.

Study	D1	D2	D3	D4	D5	Overall
Kearney et al. [16]	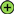	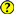	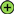	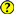	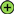	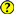
Eshoj et al. [15]	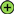	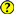	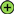	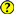	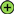	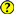
Ismail et al. [18]	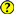	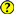	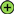	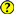	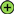	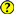
Martinez-Rico et al. [19]	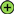	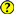	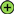	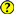	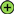	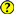

Risk of bias: 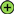 Low; 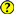 Some concerns.

**Table 3:** Risk of bias assessment according to the ROBINS-I Tool for the study by Eren et al. (2019). Domains: D1: Bias due to confounding; D2: bias in selection of participants into the study; D3: bias in classification of interventions: D4: bias due to deviations from intended interventions; D5: bias due to missing data; D6: bias in measurement of outcomes; and D7: bias in selection of the reported result.

Study	D1	D2	D3	D4	D5	D6	D7	Overall
Eren et al. [17]	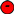	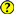	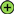	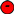	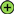	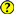	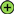	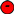

Risk of bias: 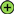 Low; 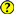 Moderate; 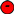 Serious.

**Table 4:** GRADE assessment of certainty of evidence for shoulder function in the two treatment conditions (conservative, post-surgical).

Treatment	Participants (Studies)	Risk of Bias	Inconsistency	Indirectness	Imprecision	Publication Bias	Evidence
Conservative	538 (2)	−1	None	−1	None	None	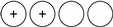 Low
Post-surgical	151 (3)	−1	None	None	None	None	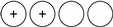 Low

## Data Availability

Dataset available on request from the authors.

## Appendix A. Search Strategy

MEDLINE (PubMed), 20 August 2024

Search Terms

(shoulder OR acromioclavicular OR sternoclavicular)

AND (dislocation OR sprain OR subluxation OR avulsion OR laceration OR hemarthrosis OR rupture OR tear OR "open wound" OR traumatic[Title/Abstract])

AND (supervised OR physiotherapy OR exercise[Title/Abstract] OR rehabilitation[Title/Abstract] OR training[Title/Abstract] OR therapy[Title/Abstract] OR guided[Title/Abstract] OR "in-person"[Title/Abstract] OR "face-to-face" OR "usual care"[Title/Abstract] OR "standard care"[Title/Abstract] OR "recommended care"[Title/Abstract])

AND (unsupervised OR home* OR tele* OR self-* OR remote OR internet OR smartphone[Title/Abstract] OR digital[Title/Abstract] OR video*[Title/Abstract])

AND (controlled OR randomized OR comparative OR "control group")

NOT ("study protocol"[Title/abstract] OR "case report"[Title] OR "case study"[Title] OR review[Title/abstract] OR arthroplasty[Title/abstract] OR cancer[Title/abstract] OR brain[Title/abstract] OR "covid"[Title])

AND ("1990/01/01"[Date - Publication]: "2024/08/01"[Date - Publication])

Total Hits: 191

## Appendix B. Summary Functional Outcomes Included Studies

**Table A1:** Functional scores of the included studies across different time points, grouped by intervention type. Baseline and post-intervention (follow-up) values are presented as means (SD). Values not reported are marked as “-”. Scores utilized in the meta-analysis are highlighted in bold.

Study	Outcome	Assessment Time	Supervised	Self-Managed
Conservative Treatment				
Eshoj et al., 2020 [15]	WOSI (total)	Baseline	970.2 (346.9)	1145.5 (376.2)
		3 Months	655.3 (-)	427.2 (-)
	**CMS**	Baseline	72.6 (15.3)	67.6 (20.7)
		**3 Months**	**56.3 (-)**	**54.6 (-)**
Kearney et al., 2024 [16]	**OSIS**	Baseline	-	-
		6 Weeks	23.3 (10.4)	24.4 (9.9)
		**3 Months**	**32.2 (10.4)**	**30.0 (11.4)**
		6 Months	38.4 (9.2)	36.2 (10.7)
		1 Year	41.6 (7.8)	39.9 (9.2)
	QuickDASH	Baseline	-	-
		6 Weeks	27.6 (21.4)	32.8 (23.2)
		3 Months	19.3 (19.9)	22.8 (21.7)
		6 Months	12.7 (16.9)	14.4 (17.5)
		1 Year	9.2 (15.2)	11.0 (16.0)
Post-surgical Treatment				
Eren et al., 2019 [17]	DASH	Baseline	32.53 (16.42)	27.46 (11.81)
		3 Months	11.08 (11.41)	7.93 (8.4)
		6 Months	5.32 (5.24)	4.44 (4.48)
		1 Year	3.16 (4.09)	1.79 (2.34)
	**CMS**	Baseline	54.17 (10.46)	58.23 (14.23)
		**3 Months**	**85.03 (17.92)**	**85.29 (14.02)**
		6 Months	92.54 (8.13)	93.27 (4.99)
		1 Year	95.97 (5.68)	96.54 (4.36)
	Rowe	Baseline	43.81 (19.16)	51.72 (15.36)
		3 Months	87.0 (12.04)	86.79 (15.64)
		6 Months	94.25 (10.16)	95.71 (4.01)
		1 Year	98.0 (4.70)	98.21 (2.79)
Ismail et al., 2014 [18]	**FIT-HaNSA**	Baseline	-	-
		**12 Weeks**	**251.5 (39.5)**	**240.3 (52.7)**
		24 Weeks	284.4 (28.9)	261.2 (57.6)
Martinez-Rico et al., 2018 [19]	DASH	Baseline	28.9 (2–54)	30.1 (31–58)
		4 Months	9.0 (0–36)	25.9 (9–86)
		6 Months	2.9 (0–27)	9.7 (0–45)
		12 Months	0.9 (0–18)	5.0 (0–45)
	**OSIS**	Baseline	36.4 (25–53)	36.7 (24–53)
		**4 Months**	**20.4 (12–36)**	**26.4 (12–51)**
		6 Months	14.5 (12–30)	21.6 (12–33)
		12 Months	12.5 (12–23)	17.2 (12–32)
	Rowe	Baseline	42.6 (15–75)	40.0 (5–75)
		12 Months	93.4 (70–100)	89.1 (65–100)

## Appendix C. Sensitivity Analysis

**Table A2:** Sensitivity analysis for studies included in the treatment mode post-surgical exercise therapy (random effects model).

Study Omitted	SMD	95% CI	*p*-Value	$\tau^{2}$	τ	$I^{2}$ (%)
Eren et al., 2019 [17]	−0.59	[−3.71;2.53]	0.25	0.03	0.17	21.9
Ismail et al., 2014 [18]	−0.38	[−5.32;4.56]	0.51	0.23	0.48	77.0
Martinez-Rico, 2018 [19]	−0.07	[−1.63;1.49]	0.66	0.00	0.00	0.0
Pooled estimate	−0.35	[−1.39;0.69]	0.28	0.11	0.33	55.7

## References

[1] World Health Organization (WHO) (2019). Dislocations, Sprains and Strains of Joints and Ligaments of the Shoulder and Upper Arm. International Statistical Classification of Diseases and Related Health Problems (ICD-10).

[2] Robinson C.M., Howes J., Murdoch H., Will E., Graham C. (2006). Functional Outcome and Risk of Recurrent Instability After Primary Traumatic Anterior Shoulder Dislocation in Young Patients. J. Bone Jt. Surg..

[3] Shah A., Judge A., Delmestri A., Edwards K., Arden N.K., Prieto-Alhambra D., Holt T.A., Pinedo-Villanueva R.A., Hopewell S., Lamb S.E. (2017). Incidence of shoulder dislocations in the UK, 1995–2015: A population-based cohort study. BMJ Open.

[4] Killian M.L., Cavinatto L., Galatz L.M., Thomopoulos S. (2012). The role of mechanobiology in tendon healing. J. Shoulder Elb. Surg..

[5] Kim S.H., Ha K.I., Jung M.W., Lim M.S., Kim Y.M., Park J.H. (2003). Accelerated rehabilitation after arthroscopic bankart repair for selected cases: A prospective randomized clinical study. Arthrosc. J. Arthrosc. Relat. Surg..

[6] Nussbaum R., Kelly C., Quinby E., Mac A., Parmanto B., Dicianno B.E. (2019). Systematic Review of Mobile Health Applications in Rehabilitation. Arch. Phys. Med. Rehabil..

[7] Hewitt S., Sephton R., Yeowell G. (2020). The Effectiveness of Digital Health Interventions in the Management of Musculoskeletal Conditions: Systematic Literature Review. J. Med. Internet Res..

[8] Littlewood C., Malliaras P., Chance-Larsen K. (2015). Therapeutic exercise for rotator cuff tendinopathy: A systematic review of contextual factors and prescription parameters. Int. J. Rehabil. Res..

[9] Gava V., Ribeiro L.P., Barreto R.P.G., Camargo P.R. (2022). Effectiveness of physical therapy given by telerehabilitation on pain and disability of individuals with shoulder pain: A systematic review. Clin. Rehabil..

[10] Gutiérrez-Espinoza H., Araya-Quintanilla F., Cereceda-Muriel C., Álvarez Bueno C., Martínez-Vizcaíno V., Cavero-Redondo I. (2020). Effect of supervised physiotherapy versus home exercise program in patients with subacromial impingement syndrome: A systematic review and meta-analysis. Phys. Ther. Sport.

[11] Higgins J.P., Savović J., Page M.J., Elbers R.G., Sterne J.A. (2019). Assessing risk of bias in a randomized trial. Cochrane Handbook for Systematic Reviews of Interventions.

[12] Moher D., Liberati A., Tetzlaff J., Altman D.G. (2009). Preferred Reporting Items for Systematic Reviews and Meta-Analyses: The PRISMA Statement. PLoS Med..

[13] Core Team R. (2024). R: A Language and Environment for Statistical Computing.

[14] Koska D. (2024). DupliCheck: Identify Duplicates from Paper Metadata, R Package Version 0.9.

[15] Eshoj H.R., Rasmussen S., Frich L.H., Hvass I., Christensen R., Boyle E., Jensen S.L., Søndergaard J., Søgaard K., Juul-Kristensen B. (2020). Neuromuscular Exercises Improve Shoulder Function More Than Standard Care Exercises in Patients With a Traumatic Anterior Shoulder Dislocation: A Randomized Controlled Trial. Orthop. J. Sport. Med..

[16] Kearney R.S., Ellard D.R., Parsons H., Haque A., Mason J., Nwankwo H., Bradley H., Drew S., Modi C., Bush H. (2024). Acute rehabilitation following traumatic anterior shoulder dislocation (ARTISAN): Pragmatic, multicentre, randomised controlled trial. BMJ.

[17] Eren İ., Canbulat N., Atalar A.C., Eren Ş.M., Uçak A., Çerezci Ö., Demirhan M. (2019). A Clinical Comparison of Home-Based and Hospital-Based Exercise Programs Following Arthroscopic Capsulolabral Repair for Anterior Shoulder Instability. J. Sport Rehabil..

[18] Ismail M., El Shorbagy K. (2014). Motions and functional performance after supervised physical therapy program versus home-based program after arthroscopic anterior shoulder stabilization: A randomized clinical trial. Ann. Phys. Rehabil. Med..

[19] Martinez-Rico S., Lizaur-Utrilla A., Sebastia-Forcada E., Vizcaya-Moreno M.F., de Juan-Herrero J. (2018). The Impact of a Phone Assistance Nursing Program on Adherence to Home Exercises and Final Outcomes in Patients Who Underwent Shoulder Instability Surgery: A Randomized Controlled Study. Orthop. Nurs..

[20] Sterne J.A.C., Savović J., Page M.J., Elbers R.G., Blencowe N.S., Boutron I., Cates C.J., Cheng H.Y., Corbett M.S., Eldridge S.M. (2019). RoB 2: A revised tool for assessing risk of bias in randomised trials. BMJ.

[21] Sterne J.A., Hernán M.A., Reeves B.C., Savović J., Berkman N.D., Viswanathan M., Henry D., Altman D.G., Ansari M.T., Boutron I. (2016). ROBINS-I: A tool for assessing risk of bias in non-randomised studies of interventions. BMJ.

[22] Posit Team (2023). RStudio: Integrated Development Environment for R.

[23] Lüdecke D. (2018). esc: Effect Size Computation for Meta Analysis, version 0.4.1.

[24] Balduzzi S., Rücker G., Schwarzer G. (2019). How to perform a meta-analysis with R: A practical tutorial. Evid. Based Ment. Health.

[25] Walter S., Yao X. (2007). Effect sizes can be calculated for studies reporting ranges for outcome variables in systematic reviews. J. Clin. Epidemiol..

[26] Hao Q., Devji T., Zeraatkar D., Wang Y., Qasim A., Siemieniuk R.A.C., Vandvik P.O., Lähdeoja T., Carrasco-Labra A., Agoritsas T. (2019). Minimal important differences for improvement in shoulder condition patient-reported outcomes: A systematic review to inform aBMJRapid Recommendation. BMJ Open.

[27] van Kampen D.A., Willems W., van Beers L.W.A.H., Castelein R.M., Scholtes V.A.B., Terwee C.B. (2013). Determination and comparison of the smallest detectable change (SDC) and the minimal important change (MIC) of four-shoulder patient-reported outcome measures (PROMs). J. Orthop. Surg. Res..

[28] MacDermid J.C., Ghobrial M., Quirion K.B., St-Amour M., Tsui T., Humphreys D., McCluskie J., Shewayhat E., Galea V. (2007). Validation of a new test that assesses functional performance of the upper extremity and neck (FIT-HaNSA) in patients with shoulder pathology. BMC Musculoskelet. Disord..

[29] Norman G.R., Sloan J.A., Wyrwich K.W. (2003). Interpretation of Changes in Health-related Quality of Life: The Remarkable Universality of Half a Standard Deviation. Med. Care.

[30] Guyatt G.H., Oxman A.D., Vist G.E., Kunz R., Falck-Ytter Y., Alonso-Coello P., Schünemann H.J. (2008). GRADE: An emerging consensus on rating quality of evidence and strength of recommendations. BMJ.

[31] Zhang B., Fang Z., Nian K., Sun B., Ji B. (2024). The effects of telemedicine on Rotator cuff-related shoulder function and pain symptoms: A meta-analysis of randomized clinical trials. J. Orthop. Surg. Res..

[32] Huang T., Zhang W., Yan B., Liu H., Girard O. (2024). Comparing Telerehabilitation and Home-based Exercise for Shoulder Disorders: A Systematic Review and Meta-analysis. Arch. Phys. Med. Rehabil..

[33] Cutts S., Prempeh M., Drew S. (2009). Anterior Shoulder Dislocation. Ann. R. Coll. Surg. Engl..

[34] Constant C.R., Murley A.H.G. (1987). A Clinical Method of Functional Assessment of the Shoulder. Clin. Orthop. Relat. Res..

[35] Kumta P., MacDermid J.C., Mehta S.P., Stratford P.W. (2012). The FIT-HaNSA Demonstrates Reliability and Convergent Validity of Functional Performance in Patients With Shoulder Disorders. J. Orthop. Sport. Phys. Ther..

[36] Gaunt B.W., Shaffer M.A., Sauers E.L., Michener L.A., McCluskey G.M., Thigpen C.A. (2010). The American Society of Shoulder and Elbow Therapists’ Consensus Rehabilitation Guideline for Arthroscopic Anterior Capsulolabral Repair of the Shoulder. J. Orthop. Sport. Phys. Ther..

[37] Minozzi S., Cinquini M., Gianola S., Gonzalez-Lorenzo M., Banzi R. (2020). The revised Cochrane risk of bias tool for randomized trials (RoB 2) showed low interrater reliability and challenges in its application. J. Clin. Epidemiol..

[38] Donkin L., Christensen H., Naismith S.L., Neal B., Hickie I.B., Glozier N. (2011). A Systematic Review of the Impact of Adherence on the Effectiveness of e-Therapies. J. Med. Internet Res..

[39] DiMatteo M.R., Giordani P.J., Lepper H.S., Croghan T.W. (2002). Patient adherence and medical treatment outcomes: A meta-analysis. Med. Care.

[40] Alexandre N.M.C., Nordin M., Hiebert R., Campello M. (2002). Predictors of compliance with short-term treatment among patients with back pain. Rev. Panam. Salud Pública.

[41] Beinart N.A., Goodchild C.E., Weinman J.A., Ayis S., Godfrey E.L. (2013). Individual and intervention-related factors associated with adherence to home exercise in chronic low back pain: A systematic review. Spine J..

[42] Borello-France D., Burgio K.L., Goode P.S., Markland A.D., Kenton K., Balasubramanyam A., Stoddard A.M. (2010). Adherence to Behavioral Interventions for Urge Incontinence When Combined With Drug Therapy: Adherence Rates, Barriers, and Predictors. Phys. Ther..

[43] Pisters M.F., Veenhof C., de Bakker D.H., Schellevis F.G., Dekker J. (2010). Behavioural graded activity results in better exercise adherence and more physical activity than usual care in people with osteoarthritis: A cluster-randomised trial. J. Physiother..

[44] Di Fabio R.P., Mackey G., Holte J.B. (1995). Disability and Functional Status in Patients With Low Back Pain Receiving Workers’ Compensation: A Descriptive Study With Implications for the Efficacy of Physical Therapy. Phys. Ther..

[45] Vermeire E., Hearnshaw H., Van Royen P., Denekens J. (2001). Patient adherence to treatment: Three decades of research. A comprehensive review. J. Clin. Pharm. Ther..

[46] Simmich J., Ross M.H., Russell T. (2024). Real-time video telerehabilitation shows comparable satisfaction and similar or better attendance and adherence compared with in-person physiotherapy: A systematic review. J. Physiother..

[47] Lang S., McLelland C., MacDonald D., Hamilton D.F. (2022). Do digital interventions increase adherence to home exercise rehabilitation? A systematic review of randomised controlled trials. Arch. Physiother..

[48] Federal Ministry of Health (2019). Digital Healthcare Act (DVG). Federal Law Gazette I. https://www.bgbl.de/xaver/bgbl/start.xav?startbk=Bundesanzeiger_BGBl&jumpTo=bgbl119s2562.pdf#__bgbl__%2F%2F*%5B%40attr_id%3D%27bgbl119s2562.pdf%27%5D__1736779244243.

